# Supplementary material for: Functional contrast across the gray-white matter boundary
Source: Nat Commun. 2025 Jul 2;16:6077. doi: 10.1038/s41467-025-61251-w (PMC12222722; doi:10.1038/s41467-025-61251-w)
Supplement: Supplementary file 1 — Supplementary Information [file 41467_2025_61251_MOESM1_ESM.pdf]

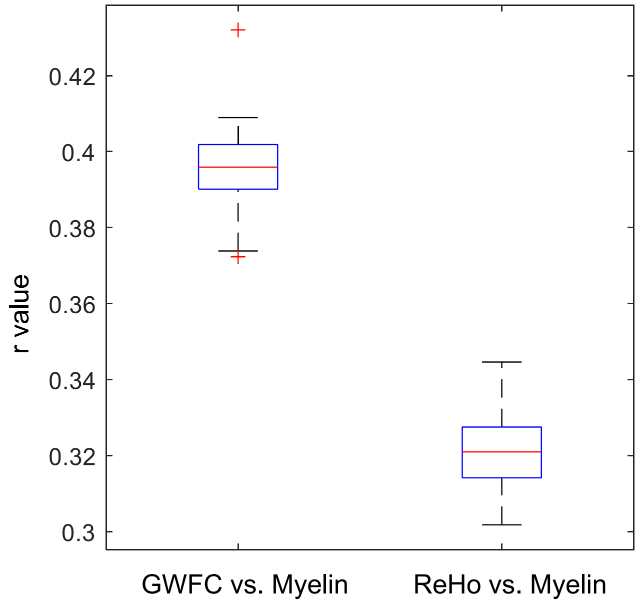

Figure S1. Comparison of correlation strength between GWFC and ReHo with myelin across 30 random subsets of subjects. To assess the robustness of the relationship between functional metrics and myelin content, we randomly selected 100 subjects (with replacement) from the full cohort (n = 687) and computed group-level GWFC and ReHo maps for each subset. For each iteration, we calculated the spatial correlation (r) between each functional metric and the cortical myelin map (across 360 regions). Boxplots summarize the distribution of r values across 30 iterations.

For each metric, the box represents the interquartile range (IQR), spanning from the 25th percentile (Q1) to the 75th percentile (Q3), with the horizontal red line inside the box indicating the median correlation value. The whiskers extend to the most extreme data points within 1.5×IQR from the box edges, and red plus symbols denote outliers beyond this range.

Abbreviations: GWFC = gray-white matter functional connectivity; ReHo = regional homogeneity.

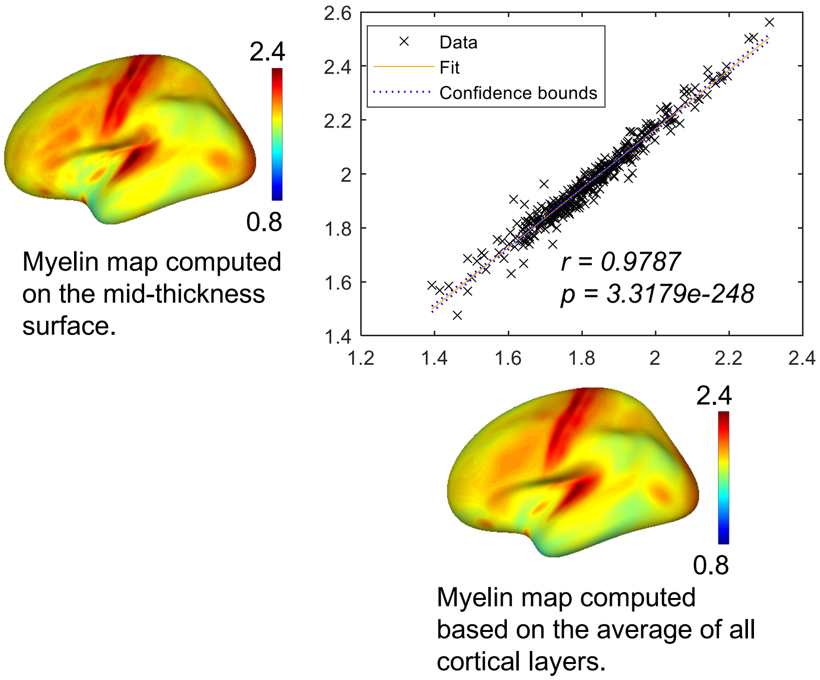

Figure S2. Comparison of myelin maps calculated using the average across all cortical layers versus those based on the mid-thickness surface. Note that the latter was computed using a subset of HCP-YA subjects (the first 100 subjects sorted by study ID). Two-sided Pearson correlation was used to assess the association between variables. The statistic is r(358) = 0.97868, p = 3.3179e-248, 95% CI = [0.97383, 0.98264]. Each point in the scatterplot represents a brain region, and the fitted line depicts the general relationship between the two measured metrics. For the distribution maps, the color represents the metric value (blue = low, red = high).

Abbreviations: HCP-Y = Human Connectome Project Young Adult Database.

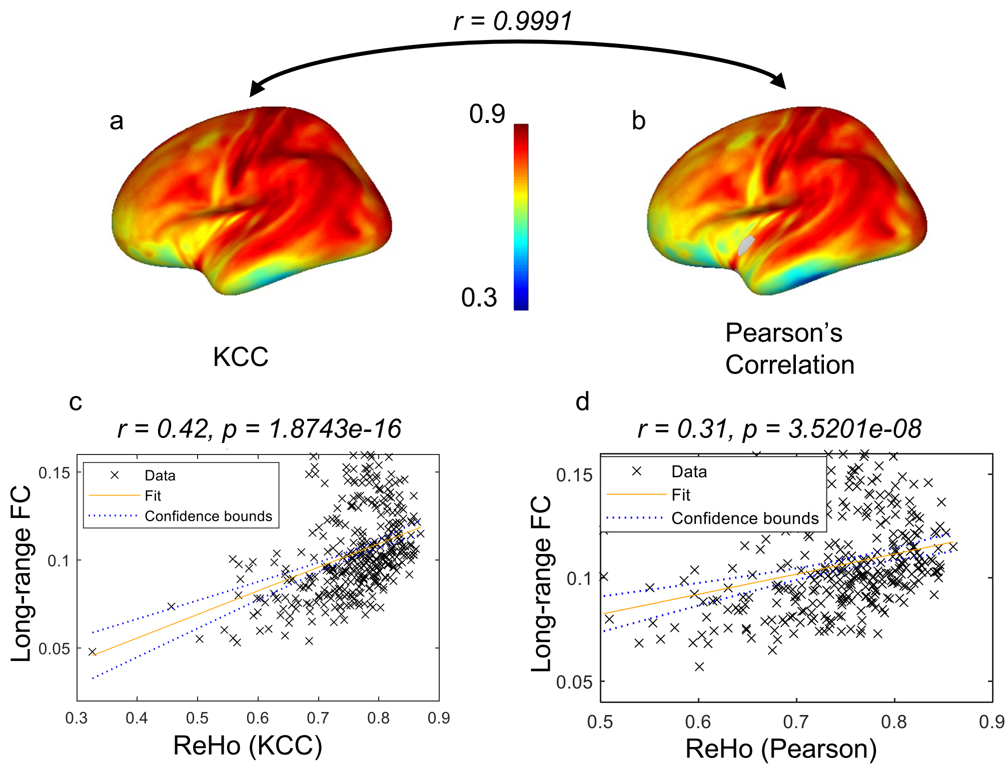

Figure S3. Comparison of ReHo Calculated Using KCC and Pearson's Correlation. Note that the maps were computed using a subset of HCP-YA subjects (the first 100 subjects sorted by study ID). Two-sided Pearson correlation was used to assess the association between variables. **(a)** shows the spatial distribution of ReHo computed using KCC, along with its corresponding relationship with long-range functional connectivity displayed in **(c)**. Their correlation is r(358) = 0.41544, p = 1.8743e-16, 95% CI = [0.32608, 0.49744]. **(b)** presents the distribution of ReHo computed using Pearson's correlation and its associated relationship with long-range functional connectivity shown in **(d)**. Their correlation is r(358) = 0.31, p = 3.5201e-08, 95% CI = [0.20, 0.41]. The spatial correlation between the two measurements is r(358) = 0.9991, p < 2.2e−16, 95% CI = [0.9989, 0.9993]. Each point in the scatterplot represents a brain region, and the fitted line depicts the general relationship between the two measured metrics. For the distribution maps, the color represents the metric value (blue = low, red = high).

Abbreviations: ReHo = regional homogeneity. KCC = Kendall's coefficient of concordance; HCP-Y = Human Connectome Project Young Adult Database.

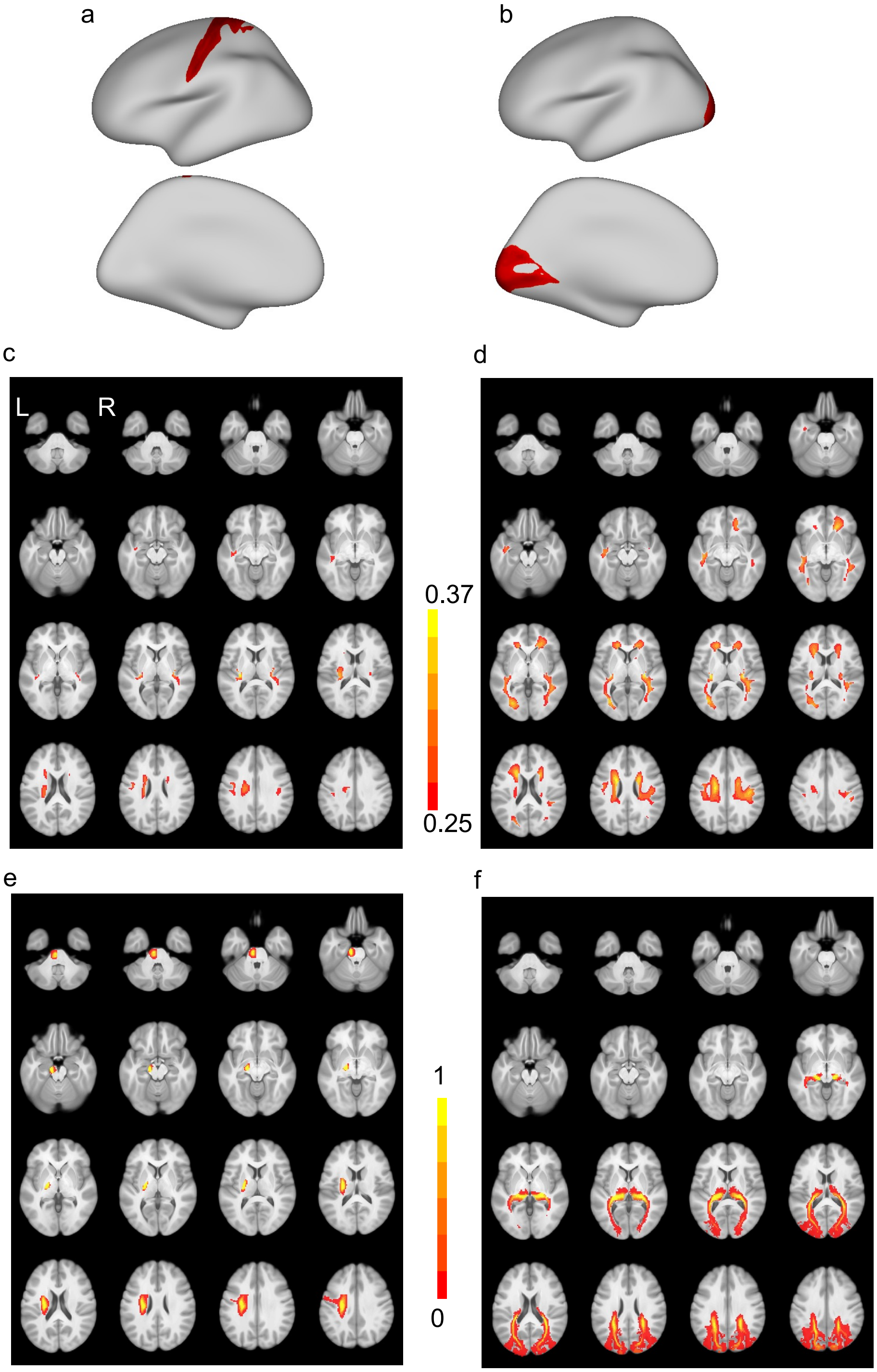

Figure S4. Long-range FC maps (r > 0.25) for two representative GM seed clusters with high GWFC values.
(a) Surface rendering of the first GM cluster.
(b) Surface rendering of the second GM cluster.
(c) Voxel-wise FC map showing correlations between all WM voxels and the GM cluster shown in (a).
(d) Voxel-wise FC map showing correlations between all WM voxels and the GM cluster shown in (b).
(e) Atlas-based distribution of corticospinal tracts.
(f) Atlas-based distribution of posterior thalamic radiations.

Color bars positioned between panels (c) and (d) indicate FC between GM seeds and WM voxels (red = low, yellow = high). Color bars positioned between panels (e) and (f) represent tract probability from the population atlas (red = low, yellow = high).

Abbreviations: FC = functional connectivity; GM = gray matter; WM = white matter; GWFC = gray-white matter functional connectivity.

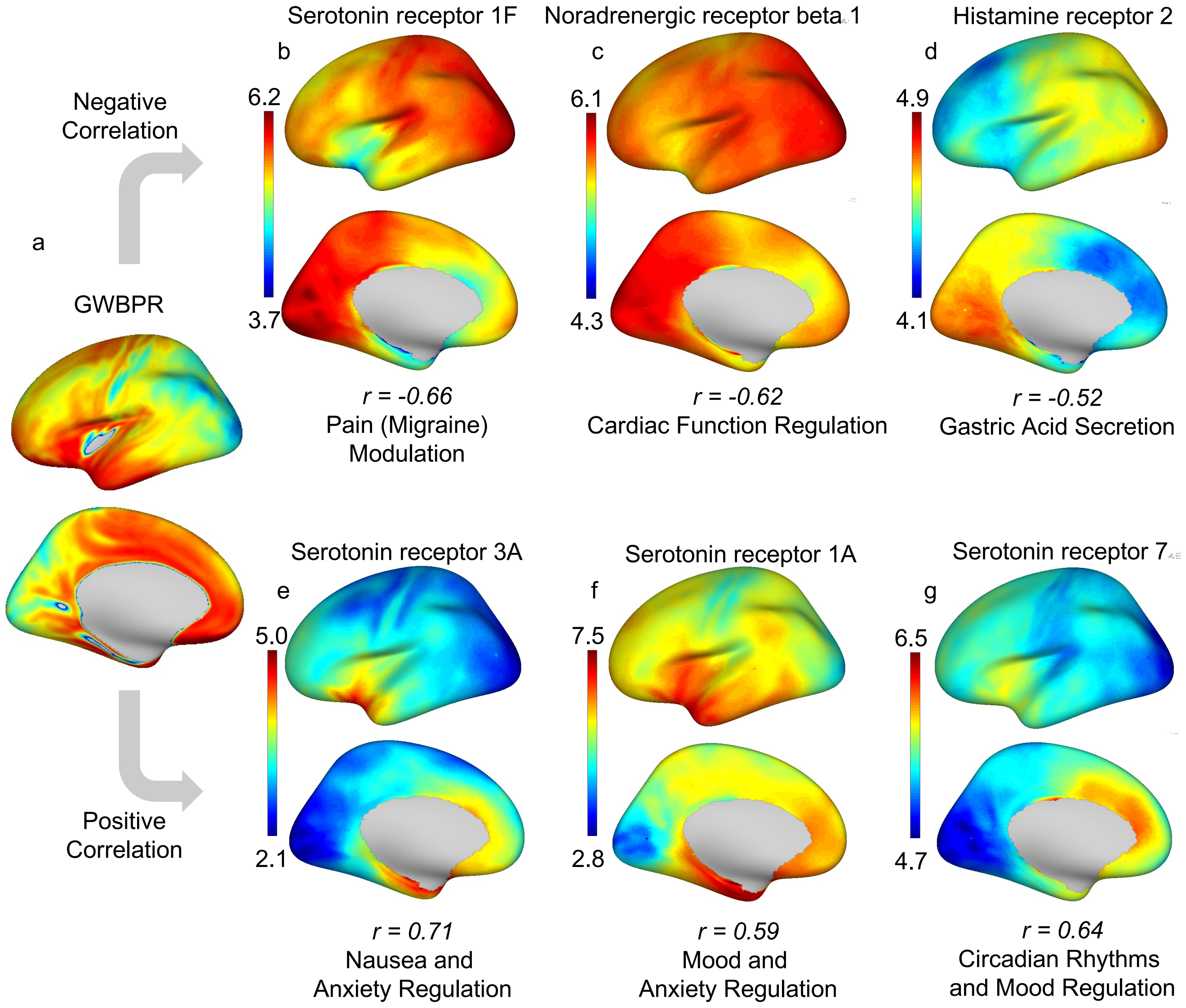

Figure S5: Correlations Between GWBPR and Neurotransmitter Receptor Distributions.
(a) The GWBPR distribution is shown across the cortex.
(b) - (d) The spatial maps display three selected neurotransmitter receptors that showed the strongest positive correlation with GWFC. However, they all show negative correlations with GWBPR.
(e) - (g) The spatial maps depict three selected neurotransmitter receptors that showed the strongest negative correlation with GWFC. However, they all show positive correlations with GWBPR.
Two-sided Pearson correlation was used to assess the association between variables. For the distribution maps, the color represents the metric value (blue = low, red = high).

Abbreviations: GWBPR = gray-white BOLD power ratio; GWFC = gray-white matter functional connectivity.

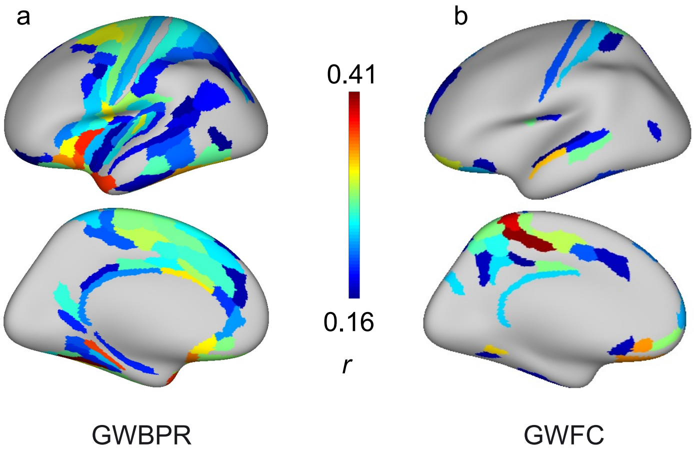

Figure S6. Significant Age-Related Variation in GWBPR and GWFC.
(a) Significant correlation between Power Ratio and Age in the HCP-D Cohort: A spatial map (ROI-wise) of the correlation between power ratio and age is presented, with warmer colors (red) of each ROI indicating stronger positive correlations.
(b) Significant correlation between GWFC and Age in the HCP-D Cohort.
Two-sided Pearson correlation was used to assess the association between variables. Regions with $p < 0.05$ are shown (The p-values have been adjusted for multiple comparisons using the Bonferroni correction).

Abbreviations: GWBPR = gray-white BOLD power ratio; GWFC = gray-white matter functional connectivity; HCP-D = Human Connectome Project Development Database; ROI = region of interest.

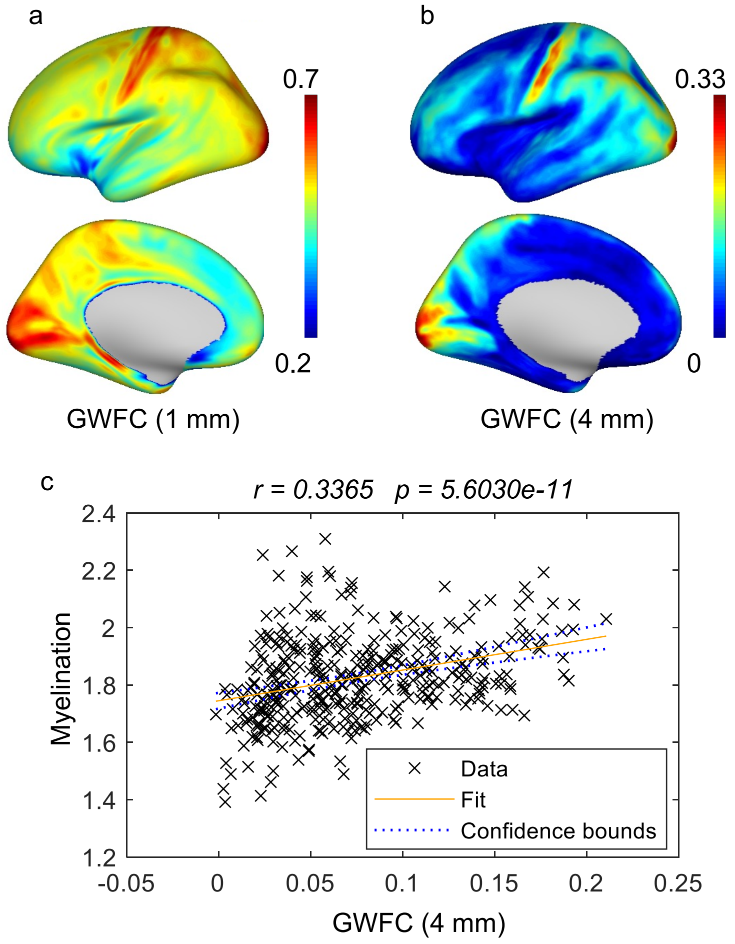

Figure S7. Comparison of GWFC maps (based on 200 subjects) calculated using WM voxels sampled at different distances from the GM-WM boundary.

(a) Spatial distribution of GWFC calculated using WM voxels located 1 mm beneath the boundary.

(b) Spatial distribution of GWFC calculated using WM voxels located 4 mm beneath the boundary.

(c) Relationship between GWFC (4 mm) and cortical myelin content (r(358) = 0.33647, p = 5.603e-11, 95% CI = [0.24151, 0.42505]), demonstrating that the spatial correlation between GWFC and myelin is preserved even when WM voxels are sampled farther from the boundary, indicating robustness against partial volume effects. Two-sided Pearson correlation was used to assess the association between variables. Each point in the scatterplot represents a brain region, and the fitted line depicts the general relationship between the two measured metrics. For the distribution maps, the color represents the metric value (blue = low, red = high).

Abbreviations: GWFC = gray-white matter functional connectivity; WM = white matter; GM = gray matter.

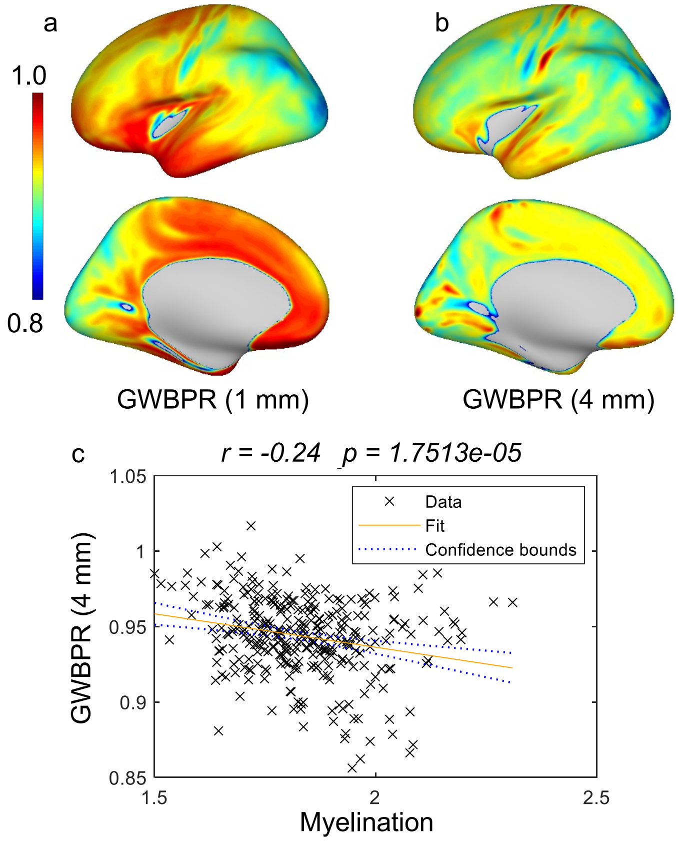

Figure S8. Comparison of GWBPR maps (based on 200 subjects) calculated using WM voxels sampled at different distances from the GM-WM boundary.

(a) Spatial distribution of GWBPR calculated using WM voxels located 1 mm beneath the boundary.

(b) Spatial distribution of GWBPR calculated using WM voxels located 4 mm beneath the boundary.

(c) Relationship between GWBPR (4 mm) and cortical myelin content (r(358) = -0.23828, p = 1.7513e-05, 95% CI = [-0.33937, -0.13175]), demonstrating that the spatial correlation between GWBPR and myelin is preserved even when WM voxels are sampled farther from the boundary, indicating robustness against partial volume effects. Two-sided Pearson correlation was used to assess the association between variables. Each point in the scatterplot represents a brain region, and the fitted line depicts the general relationship between the two measured metrics. For the distribution maps, the color represents the metric value (blue = low, red = high).

Abbreviations: GWBPR = gray-white BOLD power ratio; WM = white matter; GM = gray matter.
