# Supplementary material for: Functional contrast across the gray-white matter boundary
Source: Nat Commun. 2025 Jul 2;16:6077. doi: 10.1038/s41467-025-61251-w (PMC12222722; doi:10.1038/s41467-025-61251-w)
Supplement: Supplementary file 2 — Reporting Summary [file 41467_2025_61251_MOESM2_ESM.pdf]

nature portfolio

Corresponding author(s): Muwei Li

Last updated by author(s): Jun 10, 2025

Reporting Summary

Nature Portfolio wishes to improve the reproducibility of the work that we publish. This form provides structure for consistency and transparency in reporting. For further information on Nature Portfolio policies, see our Editorial Policies and the Editorial Policy Checklist.

Statistics

For all statistical analyses, confirm that the following items are present in the figure legend, table legend, main text, or Methods section.

n/a	Confirmed	
☐	☒	The exact sample size (*n*) for each experimental group/condition, given as a discrete number and unit of measurement
☐	☒	A statement on whether measurements were taken from distinct samples or whether the same sample was measured repeatedly
☐	☒	The statistical test(s) used AND whether they are one- or two-sided<br>*Only common tests should be described solely by name; describe more complex techniques in the Methods section.*
☐	☒	A description of all covariates tested
☐	☒	A description of any assumptions or corrections, such as tests of normality and adjustment for multiple comparisons
☐	☒	A full description of the statistical parameters including central tendency (e.g. means) or other basic estimates (e.g. regression coefficient) AND variation (e.g. standard deviation) or associated estimates of uncertainty (e.g. confidence intervals)
☐	☒	For null hypothesis testing, the test statistic (e.g. *F*, *t*, *r*) with confidence intervals, effect sizes, degrees of freedom and *P* value noted<br>*Give P values as exact values whenever suitable.*
☒	☐	For Bayesian analysis, information on the choice of priors and Markov chain Monte Carlo settings
☒	☐	For hierarchical and complex designs, identification of the appropriate level for tests and full reporting of outcomes
☐	☒	Estimates of effect sizes (e.g. Cohen's *d*, Pearson's *r*), indicating how they were calculated

Our web collection on statistics for biologists contains articles on many of the points above.

Software and code

Policy information about availability of computer code

Data collection	The data involved in this research are publicly available from Human Connectome Project database. The authors did not collect any new data involving human participants.
Data analysis	The custom code used in this study has been made available on GitHub: https://github.com/geyerou/Gray-White-Matter-Boundary.<br>Other software and toolboxes that are required for running the code include:<br>DPABI: http://rfmri.org/DPABI<br>GIFTI: https://www.nitrc.org/projects/gifti/<br>HCP workbench: https://www.humanconnectome.org/software/connectome-workbench

For manuscripts utilizing custom algorithms or software that are central to the research but not yet described in published literature, software must be made available to editors and reviewers. We strongly encourage code deposition in a community repository (e.g. GitHub). See the Nature Portfolio guidelines for submitting code & software for further information.

Data

Policy information about availability of data

All manuscripts must include a data availability statement. This statement should provide the following information, where applicable:

- Accession codes, unique identifiers, or web links for publicly available datasets
- A description of any restrictions on data availability
- For clinical datasets or third party data, please ensure that the statement adheres to our policy

The MRI data used in this study are available in the HCP database https://www.humanconnectome.org/
The source data used for generating the Figures in this study are provided in the Supplementary Information/Source Data file.

Research involving human participants, their data, or biological material

Policy information about studies with human participants or human data. See also policy information about sex, gender (identity/presentation), and sexual orientation and race, ethnicity and racism.

Reporting on sex and gender	Sex and gender were reported in the paper but were not included as variables in the analysis because they were not relevant to the study objectives.
Reporting on race, ethnicity, or other socially relevant groupings	The dataset used in this study was obtained from the Human Connectome Project (HCP). While race and ethnicity information was recorded, access to it was restricted. Additionally, it was not considered a variable in our analysis as it was not relevant to our study objectives.
Population characteristics	The dataset used in this study was obtained from the Human Connectome Project (HCP), including data from the HCP Young Adult (HCP-Y) and HCP Development (HCP-D) datasets. The HCP-Y dataset consists of 687 participants (335 males, 352 females) aged 22–35 years, while the HCP-D dataset includes 571 participants (259 males, 312 females) aged 8–21 years. All participants were screened for major neurological or psychiatric disorders, ensuring a healthy study cohort. No additional genotypic or clinical diagnosis data were used in this study.
Recruitment	Participants were recruited as part of the Human Connectome Project (HCP), specifically from the HCP Young Adult (HCP-Y) and HCP Development (HCP-D) datasets. Recruitment was conducted by the Washington University in St. Louis and involved community-based sampling. Detailed recruitment procedures followed the guidelines set by the HCP consortium.
Ethics oversight	Washington University in St. Louis

Note that full information on the approval of the study protocol must also be provided in the manuscript.

Field-specific reporting

Please select the one below that is the best fit for your research. If you are not sure, read the appropriate sections before making your selection.

☒ Life sciences ☐ Behavioural & social sciences ☐ Ecological, evolutionary & environmental sciences

For a reference copy of the document with all sections, see nature.com/documents/nr-reporting-summary-flat.pdf

Life sciences study design

All studies must disclose on these points even when the disclosure is negative.

Sample size	The dataset used in this study was obtained from the Human Connectome Project (HCP), including data from the HCP Young Adult (HCP-Y) and HCP Development (HCP-D) datasets. The HCP-Y dataset consists of 687 participants (335 males, 352 females) aged 22–35 years, while the HCP-D dataset includes 571 participants (259 males, 312 females) aged 8–21 years. We selected the maximum number of subjects available with 3T MRI and physiological recordings. This same set of MRI images was used in our previous work (https://doi.org/10.1016/j.neuroimage.2024.120887), where it demonstrated strong statistical power and high reproducibility, supporting the adequacy of the sample size for the current study.
Data exclusions	We selected 687 out of 1200 entries from the Human Connectome Project Young Adult (HCP-Y) repository and 571 out of 725 subjects from HCP development (HCP-D) repository, adhering to criteria that included the completeness of a 3T scan and associated physiological data, along with adequate data quality.
Replication	We applied our methods separately to the HCP-Y and HCP-D datasets, and the results demonstrated high reproducibility based on visual inspection, as shown in Figure 8a. Furthermore, in our supplementary analysis, we computed the proposed metrics using data subsets, and the results showed good reproducibility as quantified by Pearson's correlation.
Randomization	We did not perform any statistical analysis for inter-group comparisons; therefore, randomization is not applicable.
Blinding	We did not perform any statistical analysis for inter-group comparisons; therefore, Blinding is not applicable.

Reporting for specific materials, systems and methods

We require information from authors about some types of materials, experimental systems and methods used in many studies. Here, indicate whether each material, system or method listed is relevant to your study. If you are not sure if a list item applies to your research, read the appropriate section before selecting a response.

Materials & experimental systems

n/a	Involved in the study
☒	☐ Antibodies
☒	☐ Eukaryotic cell lines
☒	☐ Palaeontology and archaeology
☒	☐ Animals and other organisms
☒	☐ Clinical data
☒	☐ Dual use research of concern
☒	☐ Plants

Methods

n/a	Involved in the study
☒	☐ ChIP-seq
☒	☐ Flow cytometry
☐	☒ MRI-based neuroimaging

Plants

Seed stocks	N/A. This "Plant" section can not be removed from this form.
Novel plant genotypes	N/A
Authentication	N/A

Magnetic resonance imaging

Experimental design

Design type	Resting state
Design specifications	N/A
Behavioral performance measures	N/A

Acquisition

Imaging type(s)	Functional
Field strength	3T
Sequence & imaging parameters	For HCP-Y, the imaging protocols are detailed elsewhere and were performed with 3T Siemens Skyra scanners. Resting-state scans were acquired using multiband gradient-echo EPI sequences. Each session consisted of two runs of scans with opposing phase encoding directions and lasted 14 minutes and 33 seconds, with parameters TR = 720 ms, TE = 33.1 ms, and an isotropic voxel resolution of 2 mm, totaling 1200 volumes. Concurrent recordings of physiological responses, such as respiration and heartbeat, were captured during fMRI scans. Additionally, T1-weighted images were obtained using a single-echo MPRAGE sequence with a TR of 2,400 ms, TE of 2.14 ms, and voxel dimensions of 0.7 mm isotropic. T2-weighted images were obtained using a 3D T2-SPACE sequence with a TR of 3200 ms, TE of 565 ms, and voxel dimensions of 0.7 mm isotropic.<br>For HCP-D, The imaging protocols are more thoroughly described elsewhere 38. Briefly, scans were executed on Siemens 3T Prisma scanners with 32-channel head coils. The resting-state fMRI protocol involved four runs with opposing phase encoding directions, each 6 minutes and 41 seconds, with TR = 800 ms, TE = 37 ms, voxel dimension = 2 mm isotropic, and a total of 488 volumes for each run, while physiological parameters were also documented. T1-weighted images were obtained using a multi-echo MPRAGE sequence, with a TR of 2,500 ms, TEs of 1.8/3.6/5.4/7.2 ms, and voxel dimensions of 0.8 mm.
Area of acquisition	whole brain
Diffusion MRI	☐ Used ☒ Not used

Preprocessing

Preprocessing software	The data downloaded from HCP has been preprocessed. We performed further processing steps included regressing out confounding variables including 12 head movement parameters and physiological fluctuations, modeled by the RETROICOR technique 43. This preceded the application of linear trend corrections and temporal filtering using a band-pass filter covering the frequency range of 0.01–0.1 Hz.
Normalization	Images were nonlinearly nonlinearly coregistered to MNI space using FNIRT.
Normalization template	default template in FSL.
Noise and artifact removal	The fMRI processing encompassed the removal of head movement artifacts, correction of distortions from susceptibility effects using FSL based on the two runs of data with opposite-phase encoding directions, and then nonlinear registration to MNI space. Further processing steps included regressing out confounding variables including 12 head movement parameters and physiological fluctuations, modeled by the RETROICOR technique.
Volume censoring	N/A

Statistical modeling & inference

Model type and settings	We analyzed resting-state data instead of task data. Therefore this part is not applicable.
Effect(s) tested	N/A

Specify type of analysis: ☐ Whole brain ☒ ROI-based ☐ Both

Anatomical location(s): multi-modal parcellation (MMP) 1.0 atlas, each map was segmented into 360 regions (180 per hemisphere), thereby converting the map into a vector of length 360, with each element corresponding to the mean measurement within a region.

Statistic type for inference (See Eklund et al. 2016)	N/A
Correction	Bonferroni

Models & analysis

n/a	Involved in the study
☐	☒ Functional and/or effective connectivity
☒	☐ Graph analysis
☐	☒ Multivariate modeling or predictive analysis

Functional and/or effective connectivity	Pearson correlation
Multivariate modeling and predictive analysis	Within the HCP-D group, a region-by-region analysis of the change in power ratio with age was conducted using Pearson's correlation, significant correlations were found based on $p < 0.05$, Bonferroni correction.
